# How children understand aha‐experiences in problem solving

**DOI:** 10.1111/bjdp.12565

**Published:** 2025-04-17

**Authors:** Josefine Haugen, Mathilde H. Prenevost, Ida B. R. Nilsen, Evalill Bølstad, Francisco Pons, Rolf Reber

**Affiliations:** ^1^ Department of Psychology University of Oslo Oslo Norway; ^2^ Centre for Epidemic Interventions Research, Norwegian Institute of Public Health Oslo Norway; ^3^ Department of Psychology Lancaster University Lancaster UK; ^4^ Department of Psychology Stockholm University Stockholm Sweden

**Keywords:** aha‐experiences, emotion development, insight, problem solving

## Abstract

Two studies explore how 4–8‐year‐old children develop an understanding of aha‐experiences. Study 1 used a scenario approach to investigate children's understanding of the impact that having an insight has on affect. Children (*N* = 125) rated affect of a story character at different timepoints in problem‐solving scenarios with and without aha‐moments. Study 2 presented children (*N* = 167) with a story character displaying an aha response and two different stories of problem solving that may have led to the response. Results show that from age 4, children associate aha‐experiences with positive affect. However, age differences were observed for triggers of aha‐experiences. While 4‐5‐year‐olds attributed aha‐experiences to external triggers (the solution), 7–8‐year‐olds attributed them to mental triggers (a new insight). These findings indicate that children's understanding of aha‐experiences develops over time, which aligns with theories of emotional development and theory of mind.

## Graphical abstract

[Correction added on 26 June 2025: Graphical Abstract has been included in this version.]

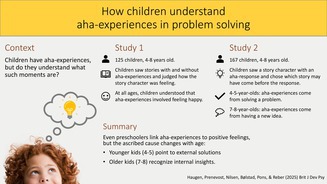

**Statement of Contribution**

**What is already known on this topic?**

- Aha‐experiences, or sudden insights, are commonly linked to positive emotions in adults.
- Children's emotional understanding and theory of mind develop significantly during early childhood.
- Prior research has shown that preschool‐aged children can begin to recognize and reason about others' emotional states and cognitive processes.

**What this study adds?**

- Children as young as 4 years old associate aha‐experiences with positive emotions.
- The study reveals developmental differences in how children attribute the causes of aha‐experiences.
  - 4–5‐year‐olds tend to link them to external events (e.g., finding a solution).
  - While 7–8‐year‐olds are more likely to attribute them to internal mental processes (e.g., having a new idea).
- These findings suggest that children's understanding of the cognitive and emotional aspects of aha‐experiences becomes more sophisticated with age, in line with established theories of emotional development and theory of mind.

Aha‐experiences consist of a sudden moment of insight, followed by metacognitive feelings including positive affect, certainty and processing fluency (Topolinski & Reber, 2010; Wiley & Danek, 2023). So far, most research has been conducted with adults. Although theoretical reasons (Prenevost & Reber, 2024) and empirical data (Haugen et al., 2024) suggest that children have insights from an early age, there is, to our knowledge, limited research on children's understanding of aha‐experiences. In the present paper, we investigate how children understand the relation between insight, affect and aha‐experiences. Gaining a better understanding of children's aha‐experiences is important because they can benefit learning through increased motivation (e.g., Skaar & Reber, 2021), improved memory (Kizilirmak et al., 2016) and a deeper understanding (Barot et al., 2024). Thus, knowledge about children's insights has implications for educational practice. Moreover, this study has theoretical implications for understanding the development of emotion understanding. To date, research has examined children's understanding of how cognitive components such as memories, expectations and beliefs influence emotion (see review Pons & Harris, 2019). The current study includes insights as an additional cognitive component.

## THE AHA‐EXPERIENCE

Aha‐experiences involve cognitive and affective elements. Insight is the cognitive part and involves a shift from a state of ignorance or impasse to a state of new understanding. It can be defined as a sudden comprehension or realization that involves the reorganization of mental representations (Kounios & Beeman, 2014). A moment of insight is often accompanied by a set of metacognitive feelings (Topolinski & Reber, 2010) which include feelings of suddenness, fluency, certainty, and positive affect.

Because the aha‐experience involves cognitive and affective processes, it is a complex phenomenon that presents an interesting case for investigating the development of children's understanding of the impact of cognition on emotion.

## CHILDREN'S UNDERSTANDING OF THE IMPACT OF COGNITION ON EMOTION

Children's understanding of how cognition influences emotions undergo several developmental changes throughout childhood (Pons & Harris, 2019). For instance, Lagattuta et al. (1997) investigated children's understanding of the effect of memory cues on emotion. When presented with illustrated vignettes where a story character sees a cue that reminds them of an emotionally loaded event, some 3‐ and 4‐year‐olds understood the links between current emotion and thinking about past events; however, only a minority were able to explain in detail how the cue triggered thoughts that in turn triggered emotion. On the other hand, most 5‐ and 6‐year‐olds successfully produced such explanations. Thus, children gradually gain the understanding that thoughts can influence feelings during the preschool years (see also de Rosnay et al., 2004; Flavell et al., 2001; Lara et al., 2019).

Of relevance to this study are two studies on how beliefs and expectations influence emotions (Asaba et al., 2019; Lara et al., 2019). The first study found that although 4‐ and 5‐year‐olds understood that a story character's expectations of a future outcome can influence their current emotions, only the 5‐year‐olds understood that expectations would continue to influence emotions after the outcome was known (Asaba et al., 2019). The second study showed that children's understanding of how expectations influence emotions depended on both the valence of the outcome and the valence of the expectation (Lara et al., 2019). For instance, 6–7‐year‐olds but not 4–5‐year‐olds understood that someone with high expectations may feel worse than someone with low expectations in the face of a negative outcome.

Taken together, the research on children's emotion understanding indicates a distinction between *external* and *mental* triggers of emotion (Pons & Harris, 2019). Children up to the age of 4–5 years increasingly understand the recognition of facial expressions and the impact of external cues and desires on emotions. From about 4–5 years to 7–8 years, children gradually develop an understanding of internal, psychological processes (such as memory and beliefs), and that these can influence emotions. This development in children's emotion understanding mirrors the development of theory of mind, with substantial gains around 4 years of age (e.g., Rakoczy, 2022; Wellman, 2010) and sustained development into middle childhood, particularly when it comes to the attribution of more complex mental states such as second‐order reflections on first‐order states (Miller, 2012, 2022). Indeed, explanations of learning as representational change (mental) instead of behavioural evidence of competence (external) correlate with the development of theory of mind (Wang & Frye, 2021).

## RECENT EVIDENCE OF CHILDREN'S UNDERSTANDING OF AHA‐EXPERIENCES

Children's understanding of aha‐experiences has only recently become a subject of research. A newly conducted study examined how young children experience and understand insight. Prenevost et al. (2025) developed an illustrated clues task inspired by the Remote Associate Test to evoke aha‐experiences. They found that the frequency of aha‐experiences remained stable across ages. However, there was a clear developmental improvement in understanding these experiences. From ages 4 to 8 years, children were increasingly likely to identify the following characteristic features of aha‐experiences: they are about a new idea, not about having an idea a person had before; they require some time (impasse), and are not immediately obvious; when they appear, they feel as though they occur suddenly, not gradually; and they come with a feeling of certainty that the idea is correct, not with a feeling of uncertainty. The only attribute of an aha‐experience that even the youngest children understood at above‐chance level was that an aha‐experience was associated with happy feelings as opposed to sad feelings. Another study suggests that children at 3 years may recognize a stereotypical aha‐gesture (raising index finger and eyebrows) and understand that it relates to cognitive processing (Mari et al., 2023).

Together, these studies provide initial evidence of children's understanding of aha‐experiences, which the present paper aims to extend.

## PRESENT STUDIES

Based on the reviewed literature, the current studies investigate how understanding of aha‐experiences develops in children.

To answer the research question, we present two independent studies. The first study uses a scenario approach to study whether young children understand the impact of having an insight on affect. The second study uses a reaction approach (Trabasso et al., 1981) to investigate how children understand that aha‐experiences might be related to problem‐solving and whether they attribute aha‐experiences to external or mental triggers. Although the studies are independent, they complement each other. While Study 1 focuses on elements that constitute an aha‐experience—namely, insight and affect—Study 2 investigates how children understand what may lead to an aha‐experience. Together, the studies provide a broader understanding of children's understanding of aha‐experiences.

Because the studies are inspired by findings from the emotion development field and due to the developmental trend discussed earlier, both studies are conducted on 4–8‐year‐old children (Lagattuta & Wellman, 2001; Lara et al., 2019; Trabasso et al., 1981).

## TRANSPARENCY AND OPENNESS

Both studies were approved by the internal ethics review board at the University of Oslo and by the Norwegian Centre for Research Data. Both studies were preregistered at AsPredicted (Study 1: #69780, Study 2: #94669). Deviations from the preregistrations, analysis strategy, and exploratory analyses are presented in the supplementary materials. The preregistration, materials, data, and analysis scripts for the studies are openly available at OSF (Study 1: https://osf.io/nfx5z/, Study 2: https://osf.io/6r5yp/). François Bernaschina at Francfort Communication & Partners designed all illustrations.

Children participated based on their parents' informed consent, obtained ahead of data collection. In addition, children could freely choose not to participate or to stop the session at any time.

The children were tested in kindergartens and schools (description of the organization of kindergartens and schools in Norway is presented in Supplementary Materials). The institutions involved in the study received small gifts (such as books or toys) as a token of appreciation for their help and efforts.

## STUDY 1

Study 1 focused on the link between insight and positive affect. This link has been documented with adults in laboratory settings (e.g., Danek et al., 2014), surveys (Skaar & Reber, 2020), and qualitative studies (Hill & Kemp, 2018). We use the term affect to denote a continuum from positive feelings to negative feelings. This terminology is commonly used in the insight literature (e.g., Topolinski & Reber, 2010).

We asked children to rate how a story character was feeling (from very happy to very sad) throughout a set of problem‐solving stories. In the stories, the character either had an insight on how to solve the problem or tried to solve the problem through trial‐and‐error. Some of the stories ended with a successful solution, whereas other stories ended with a failed attempt. This led to four story versions, combining solution type (insight/trial‐error) and solution outcome (success/failure).

Based on the reviewed evidence that young children may struggle to understand the association between thoughts and feelings, we preregistered the hypotheses that younger children would give high ratings of affect when the problem was solved (Hypothesis 1), but not at the insight moment (Hypothesis 2). While recent evidence suggests that children's understanding of the positive feelings in aha‐experiences may emerge earlier than their understanding of more abstract characteristics of aha‐experiences (Prenevost et al., 2025), it remains unclear whether children distinguish the idea (insight) and outcome (solution). That is, young children may not be able to distinguish positive affect in an insight moment—where affect is triggered by the internal thought process itself—from positive affect after successfully solving a problem, where affect is triggered by an external event. This study offers a more fine‐grained analysis of how children rate the feelings of a story character as the story unfolds.

### Method

#### Participants

We recruited 125 children aged 4–8 years without identified developmental delays. Two participants were excluded from the analysis, one because of an incomplete trial and one for not taking the tasks seriously, as reported by the experimenter in an experiment log. The final sample of 123 children was divided into 5 age groups, see Table 1.

**TABLE 1:** Age distribution table for Study 1.

Age	*N*	*N*_girls_	Mean age	Age range
4	25	15	4.51	3.89–4.98
5	32	12	5.58	5.07–5.98
6	24	12	6.46	6.07–6.87
7	24	10	7.48	7.06–7.98
8	18	9	8.43	8.04–8.92

Demographic information was not formally collected, but the participants were recruited through two schools and five kindergartens in middle‐to high‐socioeconomic‐status neighbourhoods in eastern Norway.

#### Materials

**FIGURE 1:**
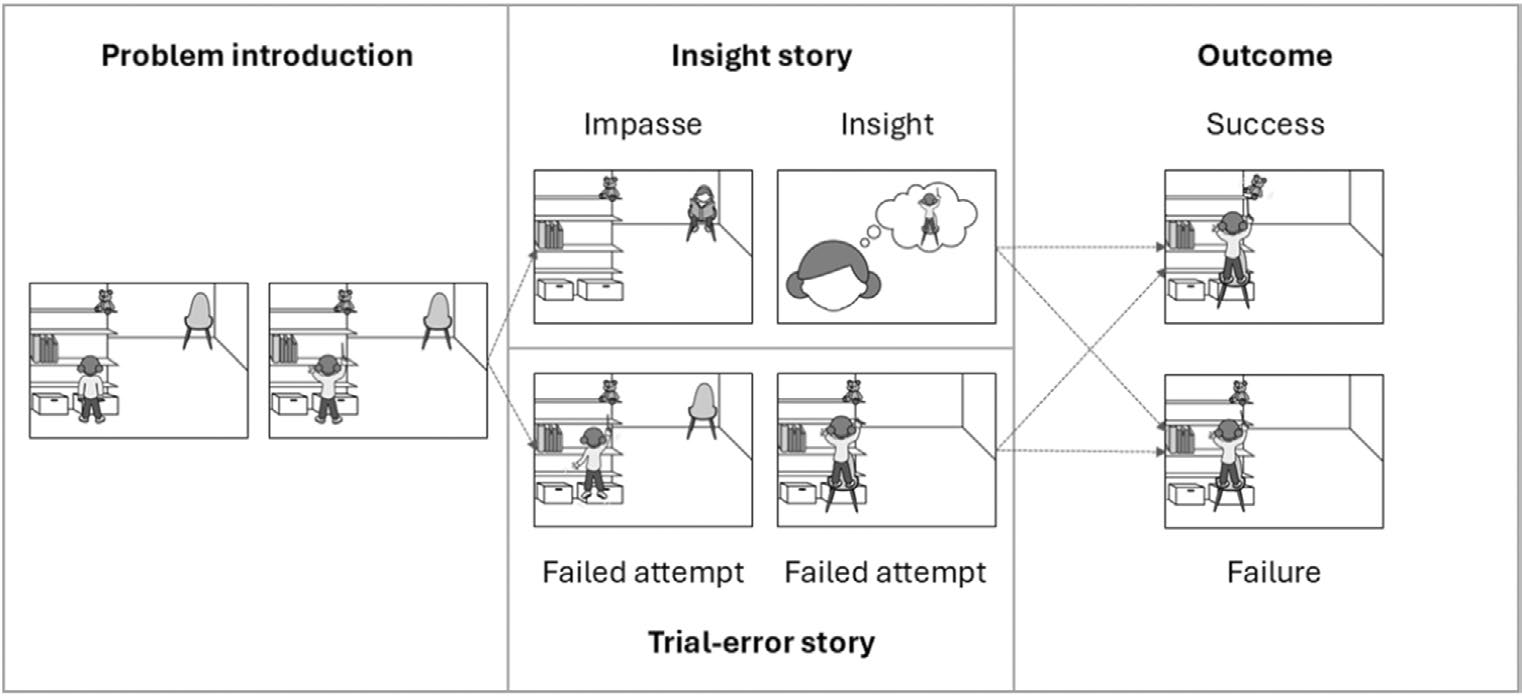
Example story illustrating the four story versions. The figure illustrates the stories used in the study. There are four story versions, combining *insight* vs. *trial‐error* stories and *successful outcome* vs. *failed outcome*. Each story follows the same basic insight sequence (e.g., Ash & Wiley, 2006). First, a problem is introduced, and a child tries to solve the problem but fails. For the insight stories, there then is an impasse, where the child stops actively trying to solve the problem, and an insight where the child has a sudden idea for a solution to the problem. Finally, at the outcome moment, the child either successfully solves the problem or tries but fails. The trial‐and‐error stories follow the same script, but instead of the impasse and insight, the child has two more failed attempts at solving the problem before the outcome, which is either successful or unsuccessful.

Story type (insight vs. trial‐error) and solution outcome (success vs. failure) were combined to yield a set of problem‐solving stories (see Figure 1).

#### Procedure

Children participated individually in a quiet room at their school or kindergarten. The experimenter welcomed the child and introduced the tasks that were presented on a laptop computer. The procedure was fully automated to reduce experimenter bias. All stories were illustrated with black‐and‐white line drawings. All prompts were audio‐recorded. The participants were asked to attribute affect to the character in the stories by choosing one out of four options on a visual rating scale. Affect judgements were collected four times for each story: First, at the end of the Introduction (Time I); second, at the Impasse moment (Time II); third, at the Insight moment (Time III); and fourth, at the Outcome moment (Time IV). Our hypotheses pertained to the latter two timepoints regarding insight and outcome. We included the first two affect judgements because we wanted to assess any unintended baseline age differences in affect judgements. The affect scale displayed facial expressions denoting very sad, sad, happy, and very happy; see Supplemental Materials for an example. To ensure that the children understood the experimental tasks, the session started with a set of practice tasks described in the Supplemental Materials that also described counterbalancing procedures.

### Results

Overall, the children's affect reflected the story versions (see Table 2). See preliminary analyses of children's affect judgements and for age differences at Time I and Time II (problem introduction) in the Supplemental Materials.

**TABLE 2:** Mean (SD) affect judgements by story version and measurement time.

Story version	Time I	Time II	Time III	Time IV
Insight with success	1.53 (0.73)	2.76 (1.07)	3.54 (0.74)	3.85 (0.48)
Insight with failure	1.42 (0.64)	2.83 (1.00)	3.58 (0.71)	1.57 (0.74)
Trial‐error with success	1.49 (0.67)	1.48 (0.69)	1.50 (0.79)	3.89 (0.46)
Trial‐error with failure	1.46 (0.62)	1.52 (0.71)	1.51 (0.82)	1.63 (0.94)

*Note*: Affect judgements: 1 = *very sad*; 2 = *a little sad*; 3 = *a little happy*; 4 = *very happy*.

#### Preregistered analyses of insight (Time III) and outcome (Time IV)

At the Insight moment (Time III), a mixed ANOVA indicated significant effects of solution type (*F* (1, 118) = 554.91, *η* ^2^_G_ = .75, *p* < .001) and age (*F* (4, 118) = 5,71, *η* ^2^_G_ = .07, *p* < .001). However, when analysed separately, the age effect was only found for trial‐error stories (*F* (4, 118) = 6.52, *η* ^2^_G_ = .18, *p* < .001), and not insight stories (*F* (4, 118) = 0.78, *η* ^2^_G_ = .03 *p* = .541). Counter to our hypothesis, all children in our sample understood that having an idea for a solution to a problem was associated with positive affect. In trial‐error stories, 4‐year‐olds gave higher ratings compared to other age groups (see Supplemental Materials).

At the outcome moment (Time IV), a mixed ANOVA found an effect of outcome (*F* (1, 118) = 994,64, *η* ^2^_G_ = .76, *p* < .001), a significant interaction for age*outcome (*F* (4, 118) = 8.28, *η* ^2^_G_ = .09, *p* < .001). In failed‐outcome stories, 4‐year‐olds gave higher ratings compared to other age groups (see Supplemental Materials). For successful outcomes, 4‐year‐olds gave lower ratings compared to 6‐year‐olds only.

#### Exploratory comparison of insight and outcome

It could be that children gave high affect ratings at the insight moment because they thought the problem was solved already. To explore this possibility, we compared affect ratings at the insight and successful outcome moments. A mixed ANOVA with age as a between‐subjects factor and time as a within‐subjects factor yielded no age effect, (*F* (4, 118) = 1.20, *η* ^2^ = .02, *p* = .314), but a significant main effect of timepoint, (*F* (1, 118) = 16.91, *η* ^2^ = .061, *p* < .001), and – most importantly – an age × time interaction, (*F* (4, 118) = 2.55, *η* ^2^ = .04, *p* = .043).

To disentangle the interaction, we calculated post‐hoc contrasts across the five age groups (Table 3). For the 4‐ and 5‐year‐olds, there were no differences in affect ratings between the insight moment and the solution moment. The differences were significant for the 6‐ and 7‐year‐olds, but not the 8‐year‐olds. Moreover, polynomial contrasts indicated a significant linear trend for age at the insight moment (*t*(118) = 3.02, *p* = .012), but not at the solution moment (*t*(118) = −0.76, *p* = .927).

**TABLE 3:** Mean affect judgements by age in stories with insight and successful outcome.

Age	Insight (Time III)	Successful outcome (Time IV)	Mean difference	df	SE	*t* ratio	*p* *
4	3.64	3.52	0.12	118	0.17	0.73	.470
5	3.66	3.91	−0.25	118	0.15	−1.71	.180
6	3.46	3.96	−0.50	118	0.17	−2.96	.015
7	3.33	3.88	−0.54	118	0.17	−3.21	.009
8	3.61	4.00	−0.39	118	0.20	−1.99	.146

*Note*: Affect judgements: 1 = *very sad*; 2 = *a little sad*; 3 = *a little happy*; 4 = *very happy*. T‐test stems from a post‐hoc comparison of the estimated mean differences between insight and successful outcome.

* *p*‐values are Holm‐Bonferroni‐corrected.

### Discussion

The children in our sample demonstrated a clear understanding that both successful outcomes and insight were associated with positive affect. The absence of age differences for the successful solutions was expected, given that previous research has demonstrated that children aged 4–5 have a good understanding of how external causes and desires may influence emotions (Pons & Harris, 2019). The absence of age differences at the insight moment was less expected. To fully understand the relationship between insights and positive affect, the child needs to understand that mental processes exist and that these may influence how people feel. Thus, at first glance, the lack of age differences in understanding of the insight‐positive affect link seems to suggest that young children may have a better understanding of the relationship between thoughts and feelings than previously thought. However, there are two alternative explanations. First, if insights are common in early childhood, the link between insight and positive affect may be familiar to young children. A recent study found that parents had observed insight moments in infants (Haugen et al., 2024). Thus, our finding that children down to preschool‐age understand that insight leads to positive affect could indicate that insight experiences are familiar to them.

Alternatively, it could be that the younger children did not distinguish between having an insight and successfully solving the problem. If the children interpreted the insight moment as though the problem was already solved, there would be no reason to assume that the story character would feel differently in these two moments. Having an insight would have the same effect on positive affect as solving a problem. This explanation is in line with both our observation that the youngest children did not differ in their judgements between Time III and Time IV and the finding that young preschoolers are less sensitive to the difference between expectations and outcomes compared to older preschoolers (Asaba et al., 2019). Study 2 addressed whether young children distinguish between having an idea for a solution to a problem and having solved a problem.

## STUDY 2

Study 2 investigates whether (1) children relate aha‐experiences to problem‐solving with new ideas and (2) attribute aha‐experiences to mental or external triggers. Hence, this study answers the question raised in Study 1 of whether children interpret the aha‐experience as though the problem was already solved. In Study 2, participants are presented with an illustration of a story character displaying an aha expression and two different narratives that could have led to this response. The participants are then asked which narrative best suits the story character's aha expression.

In line with findings on emotional development (e.g., Pons & Harris, 2019) and the findings on children's understanding of aha‐experiences provided by Prenevost et al. (2025), we predict a similar age trajectory for attributing aha‐experiences to external versus mental triggers. We predict the youngest children will perform at chance level (i.e., not score significantly above 50%) when asked to choose between problem‐solving with a new idea and an alternative story. However, as they get older, we assume a conditional probability that if the child chooses problem‐solving with a new idea, the probability that they attribute the aha‐experience to mental triggers increases with age.

### Methods

#### Participants

Children between 3 and 9 years (*N* = 167) were recruited in this study. In this study, nine 9‐year‐olds were included with the 8‐year‐olds because they had recently turned 9 years old and were in the same class as the 8‐year‐olds at school. Excluding the 9‐year‐olds did not change the results. Thus, the sample was divided into five age groups, see Table 4.

**TABLE 4:** Age distribution for Study 2.

Age	*N*	*N*_girls_	Mean age	Age range
4	30	18	4.32	3.85–4.95
5	33	14	5.59	5.05–5.99
6	36	20	6.42	6.08–6.89
7	31	12	7.47	7.0–7.93
8	37	17	8.67	8.03–9.30

*Note*: Children of age 8 and 9 were merged into one age group.

Participants were recruited from an area in Norway characterized by gentrification; therefore, the sample represented a diverse population.

Initially, we tested 174 children (the test for seven children was discontinued due to distractions). All children with a native language other than Norwegian (*N* = 15) passed the practice test (exclusion criteria) and were included.

Based on a sensitivity power analysis (see Supplementary Material), there was sufficient statistical power to detect a medium effect size for both preregistered analyses.

#### Materials

The participants were presented with four different scenarios about solving a problem. For each scenario, they answered two questions. The first question asked the participants whether they attributed aha‐experiences to suddenly having a new idea to solve a problem versus thinking of continuing an unsuccessful method to solve the same problem. The first question was used as a coarse filter to get an initial impression of whether children attributed aha‐experiences to problem‐solving with new ideas. Question 2 aimed to explain their attributions in Question 1. Was it the sudden new idea (henceforth referred to as insight) or an expectation that the problem would be resolved with the new idea (henceforth referred to as solution)? The attributions to insight versus solution align with mental triggers versus external triggers from the emotional literature. Next follows a presentation of the study material.

#### Question 1: Children's attribution of aha‐experiences to insight versus thinking of continuing to solve a problem with an unsuccessful method

**FIGURE 2:**
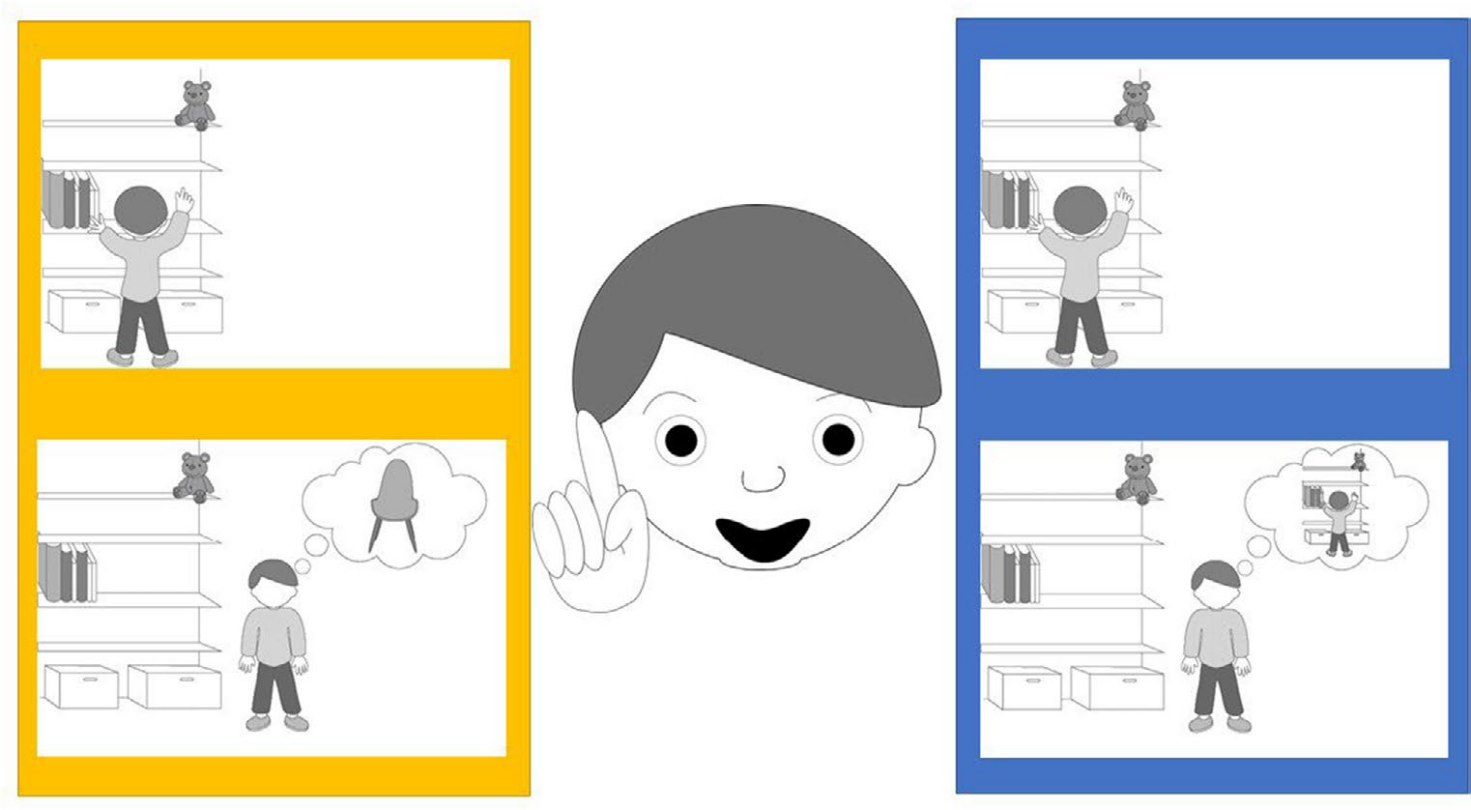
An example of illustrations to Question 1. Transcript: “Do you see the boy in the middle? He has just had an experience, and now I'll tell you what he said when it happened. “Aha!” Now, I'll tell you two stories about what the boy might have experienced. In the yellow story, you can see the boy trying to stretch his arms up to reach the teddy that is on the top shelf. He really wants to get it down, but it sits too high up. In the picture below, you see him suddenly getting an idea; maybe he can stand on a chair to reach the teddy? In the blue story, you can also see the boy trying to stretch his arms up to reach the teddy that is on the top shelf. He really wants to get it down, but it sits too high up. In the picture below, you see him thinking that he needs to continue stretching his arms to reach the teddy. Those were the two stories: Point to the story you think fits best with the boy who says aha.”

The participants first saw a line drawing of a story character pointing upward and raising eyebrows and then heard the story character exclaim, “Aha!”. Then, they were presented with two stories, each with corresponding pictures; see Figure 2 for the pictures and transcript of the spoken text to one of the scenarios. The images appeared on the screen as they were mentioned in the story.

#### Question 2: Children attributing aha‐experiences to insight or solution

**FIGURE 3:**
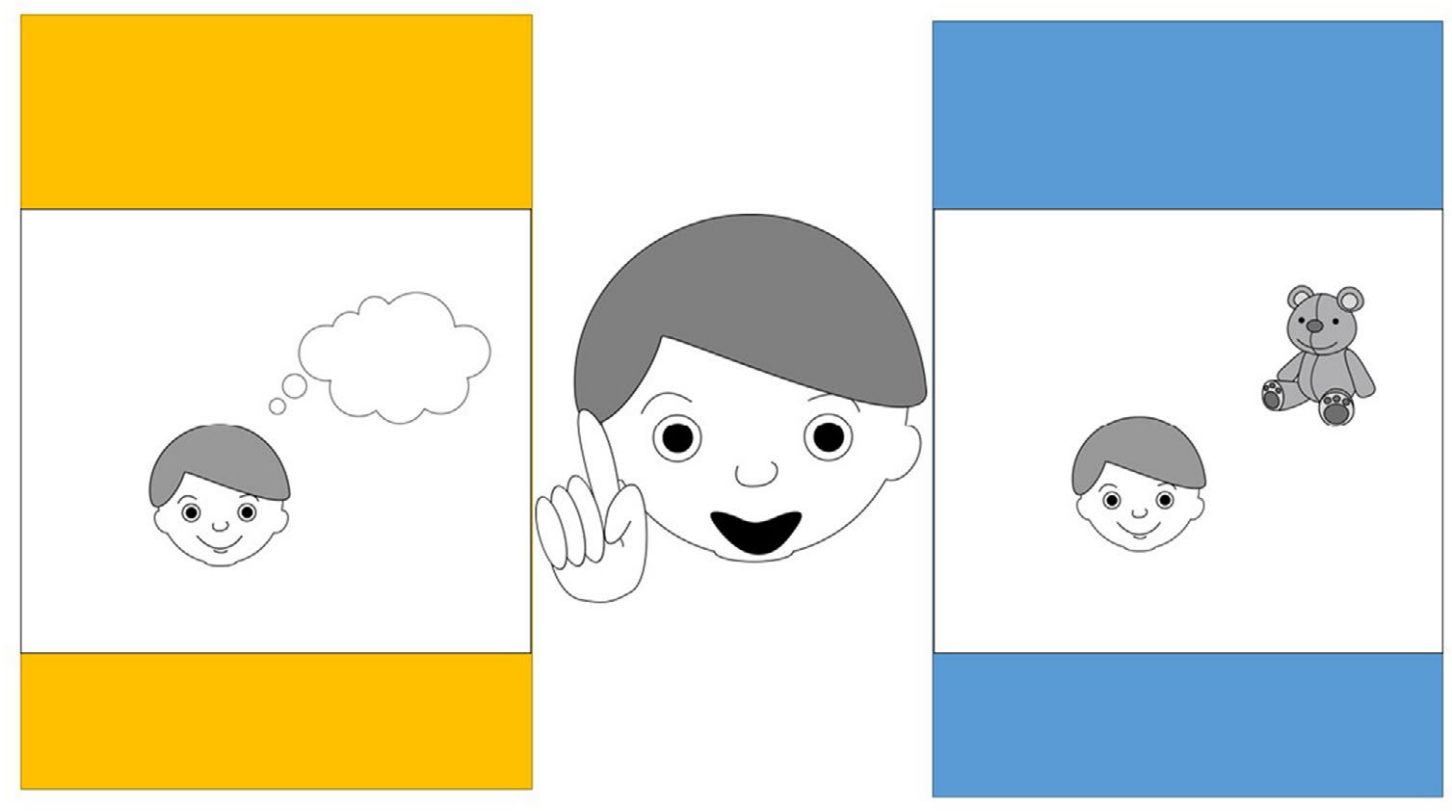
An example of illustrations to Question 2. Question 2 was conditioned on the answer in Question 1. If the participant chose “*the idea to a newly proposed solution*” in Question 1, we asked: “*Do you think the boy said aha because he is going to get the teddy down or because he suddenly got an idea? Point to the picture you think fits best*.” If the participant in Question 1 chose “*continue to do the unsuccessful method*,” we asked: “*Do you think the boy said aha because he is going to get the teddy down or because he is continuing to reach for the teddy? Point to the picture you think fits best*.”

We continued by presenting a new picture and asking a follow‐up question. Question 2 was conditioned on their answer to Question 1. The picture was the same regardless of the follow‐up questions. See Figure 3 for the picture and detailed transcript of the spoken text.

The other scenarios involved helping a cat down from a tree, finding a missing wheel for a toy car, and repairing a broken train track. All scenarios and tasks were counterbalanced for gender, background colour, and order of stories and scenarios. The materials were made using PowerPoint (Microsoft® 365), Inkscape, iMovie (version 10.3), and Audacity (version 2.3), and the test was administered using Qualtrics XM.

#### Procedure

The test condition was the same as for Study 1, except the participant provided their responses by pointing at the screen and the researchers registered the answers.

The experiment started with practice tasks, one to understand thought bubbles (same procedure as in Study 1) and another to familiarize the participant with the experimental setup (see S1 supplemental material).

### Results

Table 5 presents the distribution of probability scores for Questions 1 and 2 across different ages, with a ceiling effect observed in the older age groups for Question 1.

**TABLE 5:** Descriptive statistics and results of T‐test of children's attributions compared to chance level.

	Age	*N*	*M*	SD	df	*t*	*d*	*p*
Question 1	4	30	0.725	0.257	29	4.791	0.875	4,53E‐5*
	5	33	0.826	0.303	32	6.178	1.076	6,48E−7*
	6	36	0.888	0.193	35	12.081	2.014	4,83E−14*
	7	31	0.951	0.119	30	21.066	3.784	1,57E−19*
	8	37	0.950	0.136	36	20.310	3.340	2,61E−21*
Question 2	4	30	0.339	0.343	29	−2.576	−0.470	.015*
	5	32	0.427	0.380	31	−1.085	−0.192	.286
	6	36	0.583	0.382	35	1.308	0.218	.199
	7	31	0.777	0.309	30	4.99	0.896	<.001*
	8	37	0.709	0.366	36	3.51	0.577	.001*

*Note*: *p* (two‐tailed), Cohen's *d*, Adj. *α* = Holm‐Bonferroni correction of *α*; * means significant after correction.

#### Results Question 1

A planned polynomial contrast revealed a linear trend (*F* (1, 162) = 23.76, *η* ^2^ = .135, *p* < .001), and the results of multiple one‐sample t‐tests revealed that participants at all ages attributed the aha‐experience to the idea of a new solution significantly above chance after Holm‐Bonferroni correction (results in Table 5).

#### Results Question 2

The analyses of Question 2 were conditioned on Question 1. Only participants responding “having a new idea to solve the problem” at least once in Question 1 were included (one participant excluded). Polynomial contrast for children's attribution of aha‐experiences to insight or solution showed a linear trend (*F* (1, 161) = 30.49, *η* ^2^ = .173, *p* < .001). See S1 supplemental materials for polynomial contrast for all participants.

According to multiple one‐sample *t*‐tests of Question 2 conditioned on Question 1 (Table 5), 4‐year‐olds attributed the aha‐experience to solution; they believed that with the new idea the problem *would be solved* at above‐chance level. At ages 5 and 6, attributions were not significantly different from chance level. At ages 7 and 8, the participants attributed aha‐experiences to *insight* significantly at above‐chance level.

### Discussion

The results in Question 1 imply that children as young as 4 years old understand that aha‐experiences are related to coming up with a new idea to solve a problem over the alternative of thinking about continuing with an unsuccessful solution. This contradicts our hypothesis and is a new finding, given that the youngest children in the Prenevost et al. (2025) study did not think that an aha‐experience is about a new idea. The current study used a simpler question and showed that the youngest children understand that an aha‐experience includes a new idea. However, children's attributions become more consistent with age, as shown in Table 5. This developmental trend is in line with Prenevost et al. (2025).

Moreover, our findings showed a linear trend in children's attribution to insight. The 4‐year‐olds attributed the aha experiences to *solution* at above‐chance level. The 5‐ and 6‐year‐old children scored at chance level. Only at the age of 7 did children start to attribute the aha experience to the *insight* above chance. These results align with previous findings that children between ages 5 and 8 develop an understanding of the link between cognition and emotion and that they understand more complex emotions (Flavell et al., 2001; Lara et al., 2019; Pons & Harris, 2019). These developmental findings will be further elaborated upon in the general discussion.

The results of Question 2 did not show the same ceiling effect as Question 1 and might suggest that 8‐year‐olds did not fully understand the relationship between cognitive and the affective components of aha‐experiences. This finding is unsurprising due to previous findings that emotional development continues to progress beyond the age of 8 years (Lara et al., 2019).

## GENERAL DISCUSSION

Taken as a whole, the two studies found that: (1) young children from 4 years of age typically understood that aha‐experiences are related to positive feelings; (2) at the same age they attributed the aha‐experience to a story of an idea for a new solution to a problem significantly above chance. However, (3) while 4–5‐year‐olds attributed aha‐experiences to an external trigger, 7–8‐year‐olds attributed aha‐experiences to a mental trigger, and the 6‐year‐olds performed at chance level.

We predicted in Study 1 that younger children would not understand the relation between insight and affect in an aha‐experience. Counter to our hypothesis, there were no age differences, and children at all ages attributed high positive affect to the insight moments in the stories. Although Study 2 used a different methodological approach, the first finding in Study 2 converged with the findings from Study 1. Already from age 4, children attributed the expression of an aha‐experience to a story with a new idea. Thus, even if aha‐experiences are abstract and complex mental events, children had some understanding of aha‐experiences already at the age of 4. This is unsurprising given previous research which indicates that children at this age were able to match positive thinking with positive feelings and negative thinking with negative feelings (Bamford & Lagattuta, 2012; Lagattuta et al., 1997; Lara et al., 2019). Our findings suggested that children's understanding of the relations between thoughts, feelings, and events by 4 years of age can be applied to aha‐experiences.

One question arises from the first main findings: Was the attribution of positive emotion to insight and the attribution of an aha‐expression to having a new idea a reflection of well‐developed emotional and cognitive understanding, or did it reflect young children's limited introspection and their attribution of emotions to external triggers? Our third finding indicated that 4‐year‐olds attributed the aha‐experience to external triggers (solution), while children of the two oldest age groups associated it with mental triggers (insight). This finding aligns with theories on the development of children's emotional understanding and their development in theory of mind.

First, understanding that thoughts can influence emotions develops later than the ability to recognize how external events affect emotions (Flavell et al., 2001; Pons & Harris, 2019). This progression reflects a shift from external to internal attribution. Second, the aha‐experience is a complex phenomenon, involving both cognitive and emotional elements. Our findings suggest that children's understanding of this complex experience becomes gradually more refined but is best understood from age seven onwards. This finding followed up previous research, suggesting that during this period children begin to comprehend complex and mixed feelings. Future research may investigate aha‐experiences accompanied by negative affect, the so‐called uh‐oh moments (Hill & Kemp, 2018). Since most aha‐experiences trigger positive affect (see Skaar & Reber, 2020), it remains an open question whether uh‐oh moments like mixed emotions are understood later in childhood (Harris, 1983, 1989; Pons & Harris, 2019).

Finally, our findings were in line with the developmental trajectory of theory of mind from 4 to 8 years of age. Although children at 4 years of age already have gained some understanding of theory of mind, their understanding of the more abstract or second‐order aspects of the mind continues to develop throughout school age (Miller, 2012, 2022). In the context of Study 2, this development in theory of mind may explain why the 7‐ to 8‐year‐olds tended to attribute the aha‐experience to the new idea rather than the problem solution, because a full understanding of the aha‐experience would require the children to distinguish between the ideas of the story character and the solution to the problem. Such a correlation between explanation in terms of external versus mental attributes and development of theory of mind would be in line with earlier research on children's explanations of learning in terms of representational change (Wang & Frye, 2021). Future research may explore possibilities to accelerate understanding of an aha‐experience as an idea through theory‐of‐mind interventions that require children to explain how another person reasons about this task (see Wellman & Lagattuta, 2004).

Prenevost et al. (2025) found that 4‐ and 5‐year‐olds understood that aha‐experiences were related to positive affect, even when they did not fully grasp other aspects of aha‐experiences. Data from our Study 2 may offer an explanation. The results indicate that the youngest children recognize an aha‐experience as an idea. However, they mistakenly equate an aha‐experience with the solution. In the case of a solution, positive affect is an outcome‐related emotion (Weiner, 1985), rather than a signal indicating the discovery of a potential solution, as in an aha‐experience resulting from a new idea. Therefore, comprehending that an aha‐experience is an idea does not necessarily mean that a task is solved; it is an important developmental milestone in the understanding of aha‐experiences at around 6 to 7 years of age.

### Limitations

The present studies had several limitations to consider when interpreting the results. To capture the understanding of the youngest children, we tailored the materials specifically for 4‐year‐olds. However, it is possible that this approach had a negative impact on older children. In both studies, we observed that some 8‐year‐olds lost focus on the task because they found it easy and unstimulating. This may have led to lower performance of the older children, but it does not affect the pattern of the findings and the conclusions of the studies. Another issue regarding tailoring the materials to the youngest participants is the indication of ceiling effects, with several cases where all the children within an age group used the same response category. These ceiling effects limited the power to detect age differences in the studies and should be considered when interpreting our findings.

Moreover, we did not ask children to explain their judgements. More open‐ended questions may reveal subtle differences in children's understanding of the link between insights, feelings and aha‐experiences.

## CONCLUSION

We will conclude by summarizing our findings, providing suggestions for future directions, and highlighting possible implications. First, in line with previous research on emotion understanding, 4‐year‐old children already possessed a basic understanding that both an insightful idea about how to solve a problem and a successful outcome lead to positive feelings. Moreover, they could recognize and understand that aha‐experiences were involved in problem solving.

Second, 4‐year‐old children attributed aha‐experiences to external triggers, but with age, their understanding increased, and by age 7, children attributed aha‐experiences to the mental trigger of having an insight.

Finally, our results have implications for theory on emotion understanding, as they introduce children's understanding of aha‐experiences as a new and unexplored field. Moreover, our study has implications for theory on cognitive development and the development of insight, as it suggests that insights are familiar even to young children. In addition, as children may experience insights in educational settings, more knowledge about how children experience and understand these moments will provide new perspectives on children's motivation and learning processes.

## DECLARATION OF GENERATIVE AI AND AI‐ASSISTED TECHNOLOGIES IN THE WRITING PROCESS

During the preparation of this work, the authors used GrammarlyGo, ChatGPT and UIO GPT in order to improve the language. After using these tools, the authors reviewed and edited the content as needed and take full responsibility for the content of the publication.

## AUTHOR CONTRIBUTIONS

**Josefine Haugen:** Conceptualization; data curation; formal analysis; investigation; methodology; project administration; resources; software; supervision; validation; visualization; writing – original draft; writing – review and editing. **Mathilde H. Prenevost:** Conceptualization; methodology; software; data curation; investigation; validation; formal analysis; supervision; visualization; project administration; resources; writing – original draft; writing – review and editing. **Ida B. R. Nilsen:** Data curation; investigation; resources; software; writing – review and editing. **Evalill Bølstad:** Conceptualization; formal analysis; funding acquisition; methodology; project administration; supervision; writing – review and editing. **Francisco Pons:** Conceptualization; funding acquisition; methodology; project administration; supervision; writing – review and editing. **Rolf Reber:** Conceptualization; formal analysis; funding acquisition; methodology; project administration; supervision; validation; writing – review and editing.

## CONFLICT OF INTEREST STATEMENT

The authors declare no conflicts of interest.

## Supporting information


Data S1.

## Data Availability

The data supporting this study's findings are openly available at OSF. Study 1: https://osf.io/nfx5z/, Study 2: https://osf.io/6r5yp/.
