# Supplementary material for: How children understand aha‐experiences in problem solving
Source: Br J Dev Psychol. 2025 Apr 17;43(4):943–57. doi: 10.1111/bjdp.12565 (PMC12505822; doi:10.1111/bjdp.12565)
Supplement: Supplementary file 1 — Data S1. [file BJDP-43-943-s001.docx]

Supplemental Materials

This document contains supplemental materials for the study reported in the manuscript: "Children’s Understanding of Aha Experiences". The preregistration, materials, data and analysis scripts for the studies are openly available at OSF:

- Study 1: <https://osf.io/nfx5z/?view_only=ae6ae3f108bc45ae82f24bfad262610b>
- Study 2: <https://osf.io/6r5yp/?view_only=c5273ca2946b4b4bb44dc80f50880302>

**Table of Contents**

[Study 1 2](#_Toc187323347)

[S1a: Preregistration Deviations Table for Study 1 2](#_Toc187323348)

[S1b: Analysis plan for Study 1 3](#_Toc187323349)

[S1c: Procedure in Study 1 3](#_Toc187323350)

[S1d: Preliminary Analyses of Children’s Affect Judgements 5](#_Toc187323351)

[S1e: Preregistered Analyses of Age Differences 7](#_Toc187323352)

[Age Differences at the Insight Moment (Time III). 7](#_Toc187323353)

[Age Differences at the Outcome Moment (Time IV). 8](#_Toc187323354)

[Age Differences at Introduction (Time I) and Impasse (Time II). 9](#_Toc187323355)

[Study 2 10](#_Toc187323356)

[S2a. Analysis plan 10](#_Toc187323357)

[S2b. Sensitivity analysis 10](#_Toc187323358)

[S2c. Preregistered Exploratory Analysis 1: Kindergarten Versus School for Age 6. 11](#_Toc187323359)

[S2d. Preregistered Exploratory Analysis 2: Language 12](#_Toc187323360)

[S2e. Other Exploratory Analysis. 13](#_Toc187323361)

[References 14](#_Toc187323362)

# Study 1

## S1a: Preregistration Deviations Table for Study 1

In line with recommendations by Willroth and Atherton (2024), we report any deviations from the preregistration for the study in the table below.

| **Table S1a**  *Preregistration Deviations Table for Study 1* | | | | | |
| --- | --- | --- | --- | --- | --- |
| **Deviations** | | | | | |
| # | Details | | Original Wording | Deviation Description | Reader Impact |
| 1 | Type | Sample | We will collect data until 180 participants have completed the experiment (30 in each age group), or until January 31st 2022 (whichever comes first, provided that there are **at least 23 participants in each age group** at that date). | Although we preregistered a stopping rule of minimum 23 children per age group, only 18 8-year-olds could be recruited within the planned time frame. We had considerable challenges with recruitment related to the Covid-19 restrictions. Because of this, it was decided to go ahead with the 18 participants for the 8-year-olds.  By mistake, five 9-year-olds also participated in the study. However, they were in a different class in school than the 8-year-olds and were excluded from the analyzes because they were deemed too old to be included with the 8-year-olds, and too few to be analyzed as a separate age group. | Because there were indications of ceiling effects in this age group, it was considered acceptable to stop recruitment at the pre-registered date even if the minimum number of eight-year-olds was not met. |
|  | Reason | Plan not possible |  |  |  |
|  | Timing | During data collection |  |  |  |
| 2 | Type | Sample | Children's age will range from 4 to 8. We also include an **adult student sample.** | We had preregistered the inclusion of an adult student sample. However, due to issues with the recruitment tool during the Covid-19 pandemic which changed the participation rules for the students we did not complete data collection for the adult sample. | Again, given that ceiling effects could be observed with eight-year-olds, inclusion of adults presumably would not have changed results. |
|  | Reason | Plan not possible |  |  |  |
|  | Timing | During data collection |  |  |  |
| 3 | Type | Analysis | We will use **orthogonal contrasts** to assess the age trend for each combination of solution type and solution outcome at T3, as this is the critical time point to test our hypothesis. | Instead of orthogonal contrasts, indications of significant age differences were followed up with post-hoc pairwise Bonferroni-corrected contrasts. Given the exploratory nature of the study, we wanted to examine all combinations of age groups. | The conclusions would have remained the same if we had used orthogonal contrasts, but the results would have been less informative. |
|  | Reason | Typo/Error |  |  |  |
|  | Timing | After data access |  |  |  |
| 4 | Type | Analysis | We will use orthogonal contrasts to assess the age trend for each combination of solution type **and solution outcome** at T3, as this is the critical time point to test our hypothesis. | We did not include solution outcome in the analyses at Time III because the children had not yet seen the story outcome when they made this rating. Thus, including solution outcome would be meaningless. | In a preliminary analysis, there were no significant effects of solution outcome at Time III, so the decision of including or excluding solution outcome in this analysis did not influence our conclusions. |
|  | Reason | Typo/Error |  |  |  |
|  | Timing | After data access |  |  |  |

## S1b: Analysis plan for Study 1

The data were analysed using Analysis of Variance (ANOVA), conducted in *R* (R Core Team, 2022) with the packages *afex* (Singmann et al., 2023) and *emmeans* (Lenth, 2023). Analyses are reported in the manuscript with effect size and p-values. Effect sizes are reported with generalized eta squared (η^2^_G_), which is calculated by default by the *afex* package. Bonferroni corrections were used for post-hoc comparisons, as implemented by the *emmeans* package.

## S1c: Procedure in Study 1

To ensure that the participants understood the experimental tasks, the session started with a set of practice tasks. First, the children were familiarized with the keyboard and the keys used in the study. Next, the participants were introduced to the affect measure. The four emotion faces used in the affect-rating task were presented, and the participant was asked to indicate which face belonged with each of the four affect labels (*very happy*, *a little happy*, *a little sad*, *very sad*) by pressing the appropriate key on the keyboard. The younger children could point to the pictures on the screen and the experimenter assisted with pressing the buttons on the keyboard when necessary. Then the participant completed four practice affect tasks (two positive, two negative). Finally, thought bubbles were introduced and two practice examples were used to ensure that the participants understood that the thought bubble represents what the character is thinking about. This procedure is in line with Wellman et al. (1996), who found that children aged 3-4 could easily understand the purpose of thought bubbles in illustrated stories, given that the thought bubble had been introduced in advance. All participants had to successfully complete the practice tasks to continue to the next section.

Each child saw all four story-versions (insight with successful outcome, insight with failed outcome, trial-error with successful outcome, and trial-error with failed outcome). To reduce unintended order effects, the story order was randomized. To reduce any unintended effects related to the story content, the gender of the character in the stories and which story was presented in which of the four story-versions was counterbalanced across trials. The child shown in the affect images was the same as the character in the story. Children responded by pressing letter keys on the laptop keyboard (X for *very happy*, C for *a little happy*, V for *a little sad,* B for *very sad*).

**Figure S1b**

*Example of the Affect Rating Task*


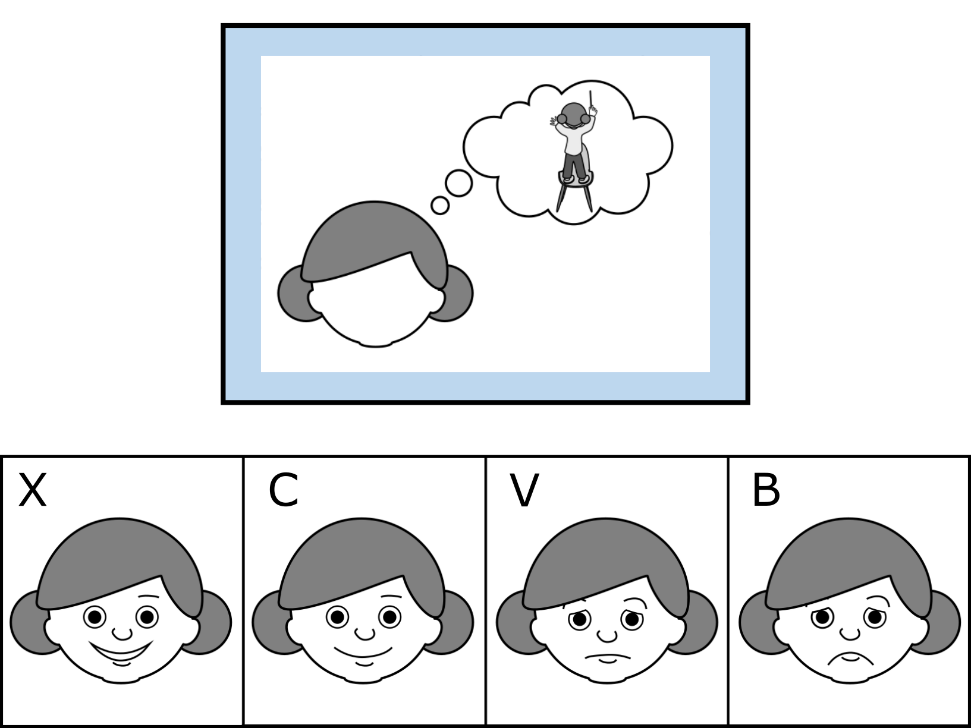


## S1d: Preliminary Analyses of Children’s Affect Judgements

Overall, the children’s affect judgements reflected the different story versions (see Table 1d-I). Preliminary analyses assessed the effect of the two experimental conditions (solution type: insight vs. trial-error; outcome: success vs. failure) and gender on the children’s affect judgements using 2 (gender) x 2 (solution type) x 2 (outcome) mixed ANOVAs.

At the first affect judgement (Time I), which was the same across all story versions, children expected the story character to feel either very sad or a little sad after an initial failed attempt to solve the problem (mean = 1.48). There were no significant effects of gender or experimental condition.

At both Time II and Time III, there was a significant effect of solution type (Time II: η^2^_G_ = .35, *p* < .001; Time III: η^2^_G_ = .64, *p* < .001). Children expected the story character to feel happier in the insight stories compared to the trial-error stories at both time points (see Table 1). Note that the affect judgements were significantly higher at Time III compared to Time II in the insight stories (mean difference = 0.76, *t*(245) = 9.97, *p* < .001). Thus, children expected the story character to feel better when they had an insight (Time III) than when they paused to do something else (Time II). There was no difference in affect judgements between Time II and Time III in the trial-error stories (mean difference = 0.004, *t*(245) = .08, *p* = .939). There was no indication of any effects of outcome or gender.

| **Table 1d-I**  *Mean (SD) Affect Judgements by Story Version and Measurement Time* | | | | |
| --- | --- | --- | --- | --- |
| **Story Version** | **Time I** | **Time II** | **Time III** | **Time IV** |
| Insight with success | 1.53 (0.73) | 2.76 (1.07) | 3.54 (0.74) | 3.85 (0.48) |
| Insight with failure | 1.42 (0.64) | 2.83 (1.00) | 3.58 (0.71) | 1.57 (0.74) |
| Trial-error with success | 1.49 (0.67) | 1.48 (0.69) | 1.50 (0.79) | 3.89 (0.46) |
| Trial-error with failure | 1.46 (0.62) | 1.52 (0.71) | 1.51 (0.82) | 1.63 (0.94) |
| *Note.* Affect judgements: 1 = *very sad*; 2 = *a little sad*; 3 = *a little happy*; 4 = *very happy*. | | | | |

At Time IV, there was a significant effect of outcome (η^2^_G_ = .74, *p* < .001). On average, children expected the story character to feel *very happy* when there was a successful outcome (*M* = 3.87, *SD* = 0.47), and between *very sad* and *a little sad* when there was a failed outcome (*M* = 1.60, *SD* = 0.85). We also found a small but significant effect of gender at Time IV (η^2^_G_ = .013, *p* = .016), and an interaction between gender and solution type (η^2^_G_ = .009, *p* = .010). Post-hoc tests indicated that in the trial-error stories, the boys gave more negative affect judgements compared to the girls (mean difference = -0.29, *t*(119) = -3.03, *p* = .003). In the insight stories there was no significant gender differences (mean difference = -0.03, *t*(119) = -0.40, *p* = .690). There was no evidence of any other effects of solution type.

| **Table S1d-II**  *Preliminary Analyses of Children’s Affect Judgements (2 x 2 x 2 mixed ANOVAs)* | | | | | | |
| --- | --- | --- | --- | --- | --- | --- |
| **DV** | **IV** | **F** | **df** | **MSE** | ***η^2^_G_*** | ***p*** |
| Affect at Time I | Gender | 0.12 | 1, 119 | 0.90 | < .001 | .726 |
|  | Solution type | 0.03 | 1, 119 | 0.36 | < .001 | .855 |
|  | Gender*Solution type | 0.59 | 1, 119 | 0.36 | < .001 | .446 |
|  | Outcome | 2.89 | 1, 119 | 0.23 | .003 | .092 |
|  | Gender*Outcome | 0.01 | 1, 119 | 0.23 | < .001 | .905 |
|  | Solution type*Outcome | 0.70 | 1, 119 | 0.30 | < .001 | .405 |
|  | Gender*Solution type*Outcome | 0.04 | 1, 119 | 0.30 | < .001 | .842 |
| Affect at Time II | Gender | <0.01 | 1, 119 | 0.98 | < .001 | .998 |
|  | **Solution type** | **179.55** | **1, 119** | **1.11** | **.348** | **< .001** |
|  | Gender*Solution type | 2.98 | 1, 119 | 1.11 | .009 | .087 |
|  | Outcome | 0.52 | 1, 119 | 0.52 | < .001 | .472 |
|  | Gender*Outcome | 2.18 | 1, 119 | 0.56 | .003 | .143 |
|  | Solution type*Outcome | 0.02 | 1, 119 | 0.50 | < .001 | .895 |
|  | Gender*Solution type*Outcome | 2.18 | 1, 119 | 0.50 | .003 | .143 |
| Affect at Time III | Gender | 0.31 | 1, 119 | 0.64 | .001 | .581 |
|  | **Solution type** | **527.55** | **1, 119** | **0.97** | **.643** | **< .001** |
|  | Gender*Solution type | 1.49 | 1, 119 | 0.97 | .005 | .225 |
|  | Outcome | 0.28 | 1, 119 | 0.39 | < .001 | .594 |
|  | Gender*Outcome | 0.45 | 1, 119 | 0.39 | < .001 | .502 |
|  | Solution type*Outcome | < 0.01 | 1, 119 | 0.40 | < .001 | .954 |
|  | Gender*Solution type*Outcome | 0.13 | 1, 119 | 0.40 | < .001 | .721 |
| Affect at Time IV | **Gender** | **5.98** | **1, 119** | **0.49** | **.013** | **.016** |
|  | Solution type | 1.05 | 1, 119 | 0.30 | .001 | .308 |
|  | **Gender*Solution type** | **6.87** | **1, 119** | **0.30** | **.009** | **.010** |
|  | **Outcome** | **789.01** | **1, 119** | **0.78** | **.737** | **< .001** |
|  | Gender*Outcome | 2.11 | 1, 119 | 0.78 | .007 | .149 |
|  | Solution type*Outcome | < 0.01 | 1, 119 | 0.28 | < .001 | .952 |
|  | Gender*Solution type*Outcome | 0.35 | 1, 119 | 0.28 | < .001 | .553 |
| *Note.* Solution type: insight vs. trial-error. Outcome: success vs. failure. *η^2^_G_*: generalized eta squared. | | | | | | |
|  | | | | | | |

## S1e: Preregistered Analyses of Age Differences

### Age Differences at the Insight Moment (Time III).

A 5 (age) x 2 (solution type) mixed ANOVA indicated significant effects of both solution type (η^2^_G_ = .75, *p* < .001) and age (η^2^_G_ = .07, *p* < .001). Moreover, there was a significant age by solution interaction (η^2^_G_ = .05 *p* = .047). To follow up on this interaction, the two story-types were analysed separately. In these analyses, the age effect could only be found in the trial-error stories (η^2^_G_ = .18, *p* < .001) but not in the insight stories (η^2^_G_ = .03 *p* = .541). In other words, counter to our hypothesis, all children in our sample understood that having an idea for a solution to a problem was associated with positive affect. When it came to the trial-error stories, post-hoc tests revealed that the 4-year-olds had significantly higher affect judgements compared to all the other age-groups (See table S1e-II). No significant differences were found among affect judgements of 5-8-year-olds.

| **Table S1e-I**  *Results of 5 (age) x 2 (solution type) mixed ANOVA at the Insight Moment (Time III)* | | | | | | |
| --- | --- | --- | --- | --- | --- | --- |
| **DV** | **IV** | **F** | **df** | **MSE** | ***η^2^_G_*** | ***p*** |
| *5 (age) x 2 (solution type) mixed ANOVA* | | | | | | |
| Affect  (Time III) | **Age** | **5.71** | **4, 118** | **0.27** | **.066** | **< .001** |
|  | **Solution type** | **554.91** | **1, 118** | **0.46** | **.749** | **< .001** |
|  | Age*Solution type | **2.49** | **4, 118** | **0.46** | **.051** | **.047** |
| *One-way ANOVA of age in insight stories* | | | | | | |
| Affect  (Time III - insight moment) | Age | 0.78 | 4, 118 | 0.36 | .026 | .541 |
| *One-way ANOVA of age in trial-error stories* | | | | | | |
| Affect  (Time III - failed attempt) | **Age** | **6.52** | **4, 118** | **0.37** | **.181** | **< .001** |
| *Note.* Solution type: insight vs. trial-error. *η^2^_G_*: generalized eta squared. | | | | | | |

| **Table S1e-II**  *Mean differences with Bonferroni-corrected post-hoc age comparisons at Time III* | | | | | | |
| --- | --- | --- | --- | --- | --- | --- |
|  | ***Trial-error stories*** | | | | | |
|  |  | **4** | **5** | **6** | **7** | **8** |
| **Insight**  **stories** | **4** | **-** | **0.489*** | **0.728***** | **0.728***** | **0.714**** |
|  | **5** | -0.029 | **-** | 0.240 | 0.240 | 0.226 |
|  | **6** | -0.003 | 0.026 | **-** | < 0.001 | -0.014 |
|  | **7** | 0.205 | 0.234 | 0.208 | **-** | -0.014 |
|  | **8** | **-**0.026 | -0.057 | -0.083 | -0.292 | - |
| *Note.* * *p < .05, ** p < .01, *** p < .001.* | | | | | | |

### Age Differences at the Outcome Moment (Time IV).

In a 5 (age) x 2 (solution type) x 2 (outcome) mixed ANOVA, there was a significant effect of outcome on affect judgements (η^2^_G_ = .76, *p* < .001), and a significant age*outcome interaction (η^2^_G_ = .09, *p* < .001). Post-hoc analyses indicated that in the stories that ended with a failed outcome, the 4-year-olds again had significantly higher affect judgements compared to all the other age groups (See table S1e-IV). In the stories with successful outcomes, only one age-contrast was significant; the 4-year-olds had more negative affect judgements compared to the 6-year-olds.

| **Table S1e-III**  *Age Effects at the Outcome Moment (Time IV) (5 x 2 x 2 mixed ANOVA)* | | | | | | |
| --- | --- | --- | --- | --- | --- | --- |
| **DV** | **IV** | **F** | **df** | **MSE** | ***η^2^_G_*** | ***p*** |
| Affect at Time IV | Age | 1.88 | 4, 118 | 0.49 | .018 | .119 |
|  | Solution type | 0.64 | 1, 118 | 0.30 | < .001 | .424 |
|  | Age*Solution type | 2.18 | 4, 118 | 0.30 | .013 | .075 |
|  | **Outcome** | **994.64** | **1, 118** | **0.63** | **.758** | **< .001** |
|  | **Age*Outcome** | **8.28** | **4, 118** | **0.63** | **.094** | **< .001** |
|  | Solution type*Outcome | 0.02 | 1, 118 | 0.28 | < .001 | .895 |
|  | Age*Solution type*Outcome | 1.07 | 4, 118 | 0.28 | .006 | .372 |
| *Note.* Solution type: insight vs. trial-error. Outcome: success vs. failure. *η^2^_G_*: generalized eta squared. | | | | | | |

| **Table S1e-IV**  *Mean differences with Bonferroni-corrected post-hoc age comparisons at Time IV* | | | | | | |
| --- | --- | --- | --- | --- | --- | --- |
|  | *Trial-error stories* | | | | | |
| **Solution type** |  | **4** | **5** | **6** | **7** | **8** |
| Insight  stories | **4** | **-** | 0.290 | 0.353 | 0.332 | **0.540**** |
|  | **5** | 0.037 | - | 0.063 | 0.042 | 0.250 |
|  | **6** | 0.115 | 0.078 | - | -0.021 | 0.188 |
|  | **7** | -0.010 | -0.047 | -0.125 | - | 0.208 |
|  | **8** | 0.030 | -0.007 | -0.086 | 0.040 | - |
|  |  | *Failure* | | | | |
| **Outcome** |  | **4** | **5** | **6** | **7** | **8** |
| Success | **4** | **-** | **0.562*** | **0.807***** | **0.619**** | **0.807***** |
|  | **5** | -0.235 | - | 0.245 | 0.057 | 0.245 |
|  | **6** | **-0.339*** | -0.104 | - | -0.188 | < 0.001 |
|  | **7** | -0.298 | -0.063 | 0.042 | - | 0.188 |
|  | **8** | -0.304 | 0.069 | 0.035 | -0.007 | - |
| *Note.* * *p < .05, ** p < .01, *** p < .001.* | | | | | | |

### Age Differences at Introduction (Time I) and Impasse (Time II).

We did not expect to see any age differences in the first two timepoints. These were included as distractors and to control for baseline age differences in affect ratings. Potential unintended age differences at these timepoints were assessed with two independent one-way ANOVAs. The results indicated that there were no effects of age at Time I (η^2^_G_ = .05, *p* = .234) or Time II (η^2^_G_ < .02, *p* = .593). Complete model summaries for all the ANOVAs can be found in the Supplemental Materials.

| **Table S1e-V**  *Analyses of Age Effects at Time I and Time II (one-way ANOVAs)* | | | | | | |
| --- | --- | --- | --- | --- | --- | --- |
| **DV** | **IV** | **F** | **df** | **MSE** | ***η^2^_G_*** | ***p*** |
| Affect at Time I | Age | 1.34 | 4, 118 | 0.22 | .043 | .261 |
| Affect at Time II | Age | 0.59 | 4, 118 | 0.24 | .019 | .674 |
| *Note.* *η^2^_G_*: generalized eta squared. | | | | | | |

# Study 2

## S2a. Analysis plan

First, we created a probability score for Question 1. Then, we created a probability score for Question 2 conditionalized on the probability of Question 1. Participants who had a score of 0 (all attributions to continuing unsuccessful solution) in Question 1 were excluded from analyses of Question 2; this applied to one 5-year-old participant.

To test our predictions, we first assessed the age trend with planned polynomial contrasts to evaluate the prediction that understanding aha experiences is related to coming up with a new idea for a solution (Question 1) and subsequent attributions to insight or solution (Question 2) increasing with age. Second, for both tasks, we analysed how participants in all age groups understood aha experiences by comparing their attributions to chance level using one-sample t-tests. We conducted Holm-Bonferroni adjustments to compensate for multiple t-tests and reduce the risk of erroneous conclusions (Holm, 1979). Data handling and analysis were performed using Excel® (Microsoft® 365), IBM SPSS (version 29), and Stata (version 17).

## S2b. Sensitivity analysis

Since there were no previously published studies on children’s understanding of aha-experiences, we did not have an approximate effect size to calculate power a priori. We therefore recruited 167 participants based on a medium effect size (d=0.5). After conducting the experiment, we performed a sensitivity analysis to assess the actual power for the polynomial contrast analysis (*η ^2^*) and the t-tests (*f*). A statistical power calculator (Linear regression power calculator, n.d.) was used to perform the calculations.

**Table S2b-I**

Overview of Statistical Power at N=167 for Eta squared and respective Cohen’s d.

| Effect Size (*η ^2^*) | Cohen's *d* | Power |
| --- | --- | --- |
| .025 | .320 | .593 |
| .05 | .459 | .838 |
| 075 | .569 | .955 |
| .125 | .756 | .998 |
| .15 | .840 | 1 |

**Table S2b-II**

Overview of Statistical Power at N=167 for *f.*

| Effect Size (*f)* | Cohen's *d* | Power |
| --- | --- | --- |
| .2 | .40 | .729 |
| .25 | .50 | .895 |
| .3 | .60 | .971 |
| .4 | .80 | .999 |
| .5 | 1 | 1 |

We concluded that with the recruitment of 167 participants, there was sufficient statistical power to detect a medium effect size for both analyses.

## S2c. Preregistered Exploratory Analysis 1: Kindergarten Versus School for Age 6.

In Norway, kindergartens offer a pedagogical approach to full-day childcare for children from their first year up to 6 years. Participation in kindergarten is voluntary, where play, social skills, and inclusivity are prioritized. The staff is highly qualified (Forskrift om rammeplan for barnehagelærerutdanning, 2012), and parents actively engage in their child’s kindergarten experience. In Norway, children start school in the fall of the year they turn six. The schoolchildren were tested at ‘AKS,’ an after-school program. It is a voluntary service at the school, available every day after regular school hours, designed to serve as an alternative learning environment that complements the school’s efforts towards students’ academic, physical, and social development.

In the present study, children aged six were recruited from both kindergartens and schools, which provide substantially different environments. Therefore, we aimed to explore possible differences between children this age in the two institutions. According to an independent t-test analysis, there were no significant differences between the six-year-old children in kindergarten and schools, neither for Question 1 (*N*_kindergarten_ = 15, *M_kindergarten_* = .90 SD*_kindergarten_* = .158; *N*_school_ = 21, *M_School_* = .88, SD_school_ = .22; *t* (34) = .288, *p* = .775, *d* = .100) nor for Question 2 (*N*_Kindergarten_ = 15, *M_kindergarten_* = .56, SD = .35; *N*_school_ = 21, *M_school_* = .60, SD = .41; *M* = .583; SD = .382; t (34) = -.364, *p* = .718, *d* =- .126).

Considering that six-year-old children in school meet higher cognitive demands than kindergarten children, the result may seem surprising. It is reasonable to expect children in school to be more exposed to metacognitive knowledge and procedures. The results may be attributed to the Norwegian school system that emphasizes a smooth transition between kindergarten and school. Therefore, the effect of schooling may not yet be detectable. Moreover, sample sizes may be too small (*N*_Kindergarten_ = 15, *N*_school_ = 21) to detect a difference, or simply that there is no effect of institution in our sample.

## S2d. Preregistered Exploratory Analysis 2: Language

We planned to include children with Norwegian as their first language and children with a different first language, so we preregistered an analysis to examine potential differences between the groups. In the event of differences, our conclusions would be based on children with Norwegian as their first language.

All children with a native language other than Norwegian passed the practice test (exclusion criteria) and were assessed by the researchers as proficient users of Norwegian. Furthermore, independent t-tests could not detect any significant differences between the groups for Question 1 (*N*_other language_ = 15, *M_other language_* = .80, *SD*_other language_ =. 194; *N_first_* _language_ = 152, *M_First_* _language_ = .88, *SD_first_* _language_ = .227; t (165) = 1.292, *p* = .198, *d* = .350) or Question 2 (*N*_other language_ = 15, *M_other language_* = .588, *SD_other language_* = .355; *N_first_* _language_ = 152, *M_first_* _language_ = .570, *SD_first_* _language_ = .394; t (165) = -.198, *p* = .844, *d* = -.054).

In this study, we could not detect a statistical difference between children with Norwegian or other languages as their first language, and all participants were included in the final statistical analysis. However, due to the small number of participants with a native language other than Norwegian (n = 15), the results of the analysis of differences should be interpreted with caution due to low statistical power.

## S2e. Other Exploratory Analysis.

**S2e-I Differences between 7 and 8-year-olds in Question 2.** Although the mean score in question 2 for the 8-year-olds (*M* = .709, *SD* = .363) was lower than for 7-year-olds *(M* = .777, *SD* = .309), there was no significant difference between the two age groups (*t* (66) = .81, *p* = .37).

**S2e-II Gender effects** There were no gender effects, neither for Question 1 (*t* (165) = .812, *d* = .126, *p* = .418) nor for Question 2 (*t* (165) = 1.768, *d* = .275, *p* = .077).

**S2e-III planned contrast not conditioned on answers in Question 1.** Some participants may have found the proposed new solution in the stories implausible or considered that continuing the unsuccessful solution might eventually work and, therefore, chose the alternative to continue with the unsuccessful solution in Question 1. We conducted the planned contrast not conditioned on answers in Question 1 to examine whether the participants still showed an increasing tendency to attribute to cognition by age. The findings remained the same. With increasing age, participants attributed aha experiences to a mental process rather than the solution (*F* (1,162) = 33.05, *η ^2^* = .187, *p* <.001)**.**

# References

Forskrift om rammeplan for barnehagelærerutdanning, LOV-2005-04-01-15-§3-2 (2012). https://lovdata.no/dokument/SF/forskrift/2012-06-04-475/%C2%A71#%C2%A71

Holm, S. (1979). A Simple sequentially rejective multiple test procedure. *Scandinavian Journal of Statistics*, *6*(2), 65–70.

Lenth, R. (2023). *Emmeans* (Version 1.8.5) [Computer software]. https://github.com/rvlenth/emmeans

*Linear regression power calculator*. (n.d.). Retrieved 19 January 2024, from https://www.statskingdom.com/33test_power_regression.html

R Core Team. (2022). *R: A language and environment for statistical computing* (Version 4.2.0) [Computer software]. R Foundation for Statistical Computing. https://www.R-project.org/

Singmann, H., Bolker, B., Westfall, J., Aust, F., & Ben-Shachar, M. (2023). *afex: Analysis of Factorial Experiments* (Version 1.2-1) [Computer software]. https://afex.singmann.science/

Wellman, H. M., Hollander, M., & Schult, C. A. (1996). Young children’s understanding of thought bubbles and of thoughts. *Child Development*, *67*(3), 768. https://doi.org/10.2307/1131860

Willroth, E. C., & Atherton, O. E. (2024). Best laid plans: A guide to reporting preregistration deviations. *Advances in Methods and Practices in Psychological Science*, *7*(1), 25152459231213802. https://doi.org/10.1177/25152459231213802
